# Hernia

**Published:** 1905-03-18

**Authors:** 


### HERNIA.

Hernia.—Taylor[1] describes a new method of radical cure of inguinal and femoral hernia. In each case the neck of the sac is approached from its abdominal aspect by a vertical incision through the parietes midway between the mid-line and the outer border of the rectus, the latter muscle being drawn inwards. The neck of the sac is isolated, cut, and tied, and the edges of the conjoined tendon and of the transversalis muscle sewn to Poupart's ligament. In the case of a femoral hernia these structures are stitched down to Cooper's ligament or to the pubic bone after holes have been drilled through the latter. Styles[2] relates the conclusions derived from 360 consecutive operations for the radical cure of hernia in infants and young children. He justifies the operation as a routine procedure by pointing out that 7 per cent. of the children attending as out-patients are cases of hernia, that in many cases in which a cure is alleged to have been brought about by a truss, the patent funicular process can still be felt by rolling the cord between the finger and thumb, that many of the most severe cases of strangulated hernia in young adults occur into sacs which have remained patent since childhood. And lastly, that if all hernias were radically cured in childhood, probably very few hernias would ever occur in young adult life. The operation consists in freeing the funicular process from the other constituents of the cord, from the tunica vaginalis, and from the tissues surrounding the internal abdominal ring. The sac is pulled down, ligatured, and cut short, when it is retracted within the abdomen without leaving any funnel-shaped opening. A single cat-gut stitch is passed through the outer pillar of the external ring, the conjoined tendon and the inner pillar. No drain is used. In children of five and upwards the legs are tied to long splints, and held extended and abducted. The penis is fixed in the neck of a glass bottle with cotton wool, and an ordinary dressing applied. In younger children no dressing but boracic and iodoform powder is used, and the legs are fixed by bandages in a position of abduction to the cot. The chest and arms are restrained and tied down by "reins," and a "cradle" keeps the bedclothes off the body. By this arrangement the urine cannot touch the wound. In the last 100 cases treated thus there was only one which suppurated. The average duration of the time the children are kept in the hospital is a fortnight. Of the 360 cases, 36 were girls, and all except one case were inguinal, the one femoral hernia occurring in a girl of eight. Of the boys, the hernia was right-sided in 75 per cent. and of the girls 66 per cent. This is probably due in boys to the late descent of the right testicle and the consequently late closure of the funicular process on that side. In the boys 78 per cent. of the hernias were observed during infancy, in girls 26 per cent. In 300 consecutive cases in boys, the exact type of sac was noticed, and in only five per cent. did it communicate with the tunica vaginalis. Sometimes a patent funicular process ran down by the side of a large tunica vaginalis, but was shut off from it. But a typical "infantile" hernia was never encountered. An hourglass contraction is often found just below the internal ring. Of the 360 cases 8·3 per cent. were "urgent" and the symptoms acute. In most of these cases there is not true strangulation but the hernia is nipped by a spasm of the conjoined tendon and will slip back if this is relieved by sleep or chloroform. In only 4·2 per cent. of the cases was it necessary to divide a constricting band before the hernia could be returned. In diagnosing a strangulated hernia in an infant there is some difficulty in distinguishing it from an acute hydrococle. Constant vomiting, constipation and suppression of urine are the indications of strangulated intestine. Strangulation is much more common in infants (under 18 months) than in older children. In 7 per cent. the cæcum occupied the hernial sac and the vermiform appendix could often be felt through the scrotum. In five out of the 36 cases in girls the sac contained the ovary and Fallopian tube. In 2 per cent. of all the cases the sac was tuberculous, many sago-like tubercles being scattered over the membrane. This can often be diagnosed before operation by the thickening of the cord, felt after the hernia has been returned. As regards results:—Only four cases were known to have recurred, and two of these were strangulated before the original operation. Five cases died, all of these being either very delicate children, in which signs of strangulation made operation imperative. The best age to choose for operation is just before teething; but of the actual operations related only 26 were performed on children under 12 months. Kennedy[3] describes a new operation for the radical treatment of inguinal hernia. He makes the usual skin incision above Poupart's ligament. The sac is then separated from the cord, and the latter is drawn down into the scrotum. The sac is freed by the finger up to the internal ring and invaginated (as in Kocher's latest method) and brought through the abdominal muscles, peritoneal surface outwards, and its base fixed there by a stitch, the rest being cut off. The edge of the conjoined tendon is next sewn to Poupart's ligament by stitches introduced by a kind of aneurism needle. The point of the latter is guided by a finger in the inguinal canal. The advantage of this method is that the aponeurosis of the external oblique is not divided. In a series of 103 operations 76 were traced from two to six years afterwards. Of these 54 treated by the author's method presented only one recurrence; eight by Bassini's method presented two recurrences; 14 by other methods (Kocher's or Macewen's) presented two recurrences. Dowden[4] describes a case of hernia into the "superior" duodenal fossa. The patient was a delicate boy of 13 who gave a history of recurrent attacks of severe abdominal pain. The last attack, which began three days before entering the hospital, was accompanied by vomiting, constipation, and collapse. A pyriform tender swelling was present to the left of the umbilicus. An incision was made to the left of the navel, and the mass proved to be an internal hernia. The coils of ileum which occupied the sac were drawn out of it without much difficulty. There were several tubercular nodules on the intestine and in the sac wall. The cavity of the sac was drawn together with a few deep stitches and the abdomen closed. Patient died a few hours later. Autopsy showed tubercular peritonitis, and a hernial sac into the superior duodenal fossa, which measured about $4\frac{1}{2}$ inches in diameter, and its mouth, which was $1\frac{1}{2}$ inch wide, looked downwards and to the left. The middle colic artery ran in front of the opening, the jejunum emerged from it, and the transverse meso-colon covered the sac.

[1] Lancet, Dec. 24, 1904. [2] Ibid., Oct. 1, 1904. [3] Ibid., Oct. 1, 1904. [4] Scottish Med. and Surg. Jour., Vol. xv., No. 1.

